# Characterization of the complete chloroplast genome of *Pterocarya stenoptera*, a tall deciduous tree of the family Juglandaceae

**DOI:** 10.1080/23802359.2020.1715874

**Published:** 2020-01-22

**Authors:** Zhongyi Yang, Yilu Wang

**Affiliations:** a College of Life Sciences, Taizhou University, Taizhou, China;; b College of Life Sciences, Shanghai Normal University, Shanghai, China

**Keywords:** *Pterocarya stenoptera*, chloroplast genome, shade tree, phylogenetic analysis

## Abstract

*Pterocarya stenoptera*, also called Chinese wingnut in China, is a tall deciduous tree of the walnut family (Juglandaceae). *P. stenoptera* is one of the most popular shade trees owing to its tall and graceful form and rapid growth rate. In the present study, the chloroplast genome of *P. stenoptera* was assembled and analyzed phylogenetically. The chloroplast genome of *P. stenoptera* is 160,212 bp in length, with a large single-copy region (LSC) of 88,724 bp, a small single-copy region (SSC) of 18,396 bp, and a pair of inverted repeat regions (IRs) of 26,046 bp, forming a typical quadripartite structure. A total of 130 genes are annotated from the chloroplast genome of *P. stenoptera*, including 82 protein-coding genes, 40 transfer RNA (tRNAs) genes, and 8 ribosomal RNA (rRNAs) genes. The GC content of the chloroplast genome is 36.2%. Phylogenetic analysis based on the common proteins from *P. stenoptera* and 14 related species confirmed the close relationship between Pterocarya and Juglans.

*Pterocarya stenoptera*, also called Chinese wingnut, is a tall deciduous tree of the walnut family Juglandaceaeis. *P. stenoptera* mainly grows along the riverbanks or mountain slopes in warm temperate and subtropical zones of China. *P. stenoptera* is one of the most popular shade trees owing to its tall and graceful form and fast growth rate (Yang et al. 2013). In addition to be used as landscape plants, *P. stenoptera* has a long history of traditional medicinal uses (Wang et al. 2006).

The chloroplast genome is highly conserved among related plant species, and has been frequently used in plant phylogenetic and biogeographical research, as well as genetic diversity research (Abla et al. 2019). Although the chloroplast genomes of many species in family Juglandaceaeis, including several species in Juglans (Dong et al. 2017), and *Platycarya strobilacea* (Yan et al. 2017) of genus Platycarya have been reported, there is no complete chloroplast genome sequence of Pterocarya species available in public database. Here, the complete chloroplast genome of *P. stenoptera* was determined, annotated, and analyzed phylogenetically.

The leaf samples of *P. stenoptera* were collected from Beijing Botanical Garden, Beijing, China (106°79′E, 39°83′N). The DNA sample (No. 20190818-03) was stored in College of Life Sciences, Taizhou University of China, Taizhou, Zhejiang Province, China. Genomic DNA was extracted from fresh leaf sample using the modified CTAB method (Doyle 1987) and sequenced using Illumina HiseqX Ten platform. Approximately 5GB clean reads (paired-end 150 bp) were generated and used for chloroplast genome assembly using NOVOPlasty v 3.7.2. (Dierckxsens et al. 2017). The assembled chloroplast genome was annotated with PGA (Qu et al. 2019). The *P. stenoptera* chloroplast genome sequence was deposited in GenBank (Accession number: MN866892).

The complete chloroplast genome of *P. stenoptera* is 160,212 bp in length, with a large single-copy region (LSC) of 88,724 bp, a small single-copy region (SSC) of 18,396 bp, and a pair of inverted repeat regions (IRs) of 26,046 bp, and forms a typical quadripartite structure. A total of 130 genes are annotated from the chloroplast genome of *P. stenoptera*, including 82 protein-coding genes, 40 transfer RNA (tRNAs) genes, and 8 ribosomal RNA (rRNAs) genes. The GC content of the chloroplast genome is 36.2%.

To investigate the evolutionary position of *P. stenoptera*, the complete chloroplast genomes of 12 species of family Juglandaceae, *Amborella trichopoda*, and *P. stenoptera*, were aligned using MAFFT V7.450 (Katoh and Standley 2013). A phylogenetic tree was constructed with MEGA X (Kumar et al. 2018) using maximum likelihood method with 1000 bootstrap repeats. The phylogenetic tree showed that *P. stenoptera* was closely related to all the nine Juglans species (Figure 1). In brief, the current study provides essential data for understanding the phylogenetic status of plant species in family Juglandaceae.

**Figure 1.**
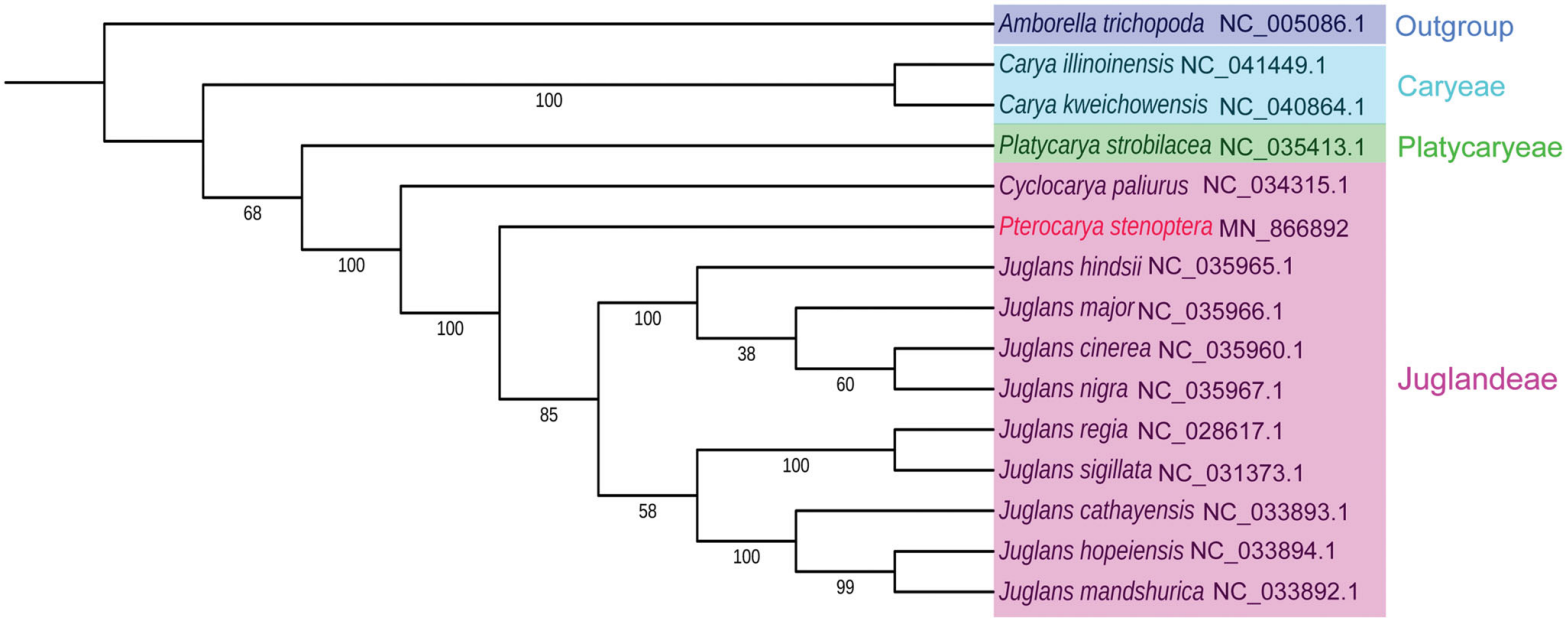
The phylogenetic tree constructed based on the 47 common proteins from the chloroplast genomes of *P. stenoptera* and other 14 plant species. Bootstrap support is indicated for each branch. All the 14 chloroplast genome sequences were downloaded from NCBI GenBank.
